# Elucidation of Differential Accumulation of 1-Phenylethanol in Flowers and Leaves of Tea (*Camellia sinensis*) Plants

**DOI:** 10.3390/molecules21091106

**Published:** 2016-08-23

**Authors:** Fang Dong, Ying Zhou, Lanting Zeng, Qiyuan Peng, Yiyong Chen, Ling Zhang, Xinguo Su, Naoharu Watanabe, Ziyin Yang

**Affiliations:** 1 Guangdong Food and Drug Vocational College, Longdongbei Road 321, Tianhe District, Guangzhou 510520, China; dongfangxyz@163.com; 2 Key Laboratory of South China Agricultural Plant Molecular Analysis and Genetic Improvement & Guangdong Provincial Key Laboratory of Applied Botany, South China Botanical Garden, Chinese Academy of Sciences, Xingke Road 723, Tianhe District, Guangzhou 510650, China; yzhou@scbg.ac.cn (Y.Z.); zenglanting@scbg.ac.cn (L.Z.); pqyuan@scbg.ac.cn (Q.P.); yychen@scbg.ac.cn (Y.C.); linzh00@126.com (L.Z.); 3 Graduate School of Science and Technology, Shizuoka University, 3-5-1 Johoku, Naka-ku, Hamamatsu 432-8561, Japan; acnwata@ipc.shizuoka.ac.jp

**Keywords:** aroma, biosynthesis, *Camellia sinensis*, 1-phenylethanol, tea, volatile

## Abstract

1-Phenylethanol (1PE) is a major aromatic volatile in tea (*Camellia sinensis*) flowers, whereas it occurs in a much smaller amounts in leaves. Enzymes involved in the formation of 1PE in plants and the reason why 1PE differentially accumulates in plants is unknown. In the present study, enzymes in the last step leading from acetophenone to 1PE were isolated from tea flowers by traditional biochemical chromatography. The two types of partially purified enzymes were proposed to be responsible for formations of (*R*)-1PE and (*S*)-1PE, respectively. Tea leaves also contained such enzymes having equivalent activities with flowers. Stable isotope labeling experiments indicated that weak transformation from l-phenylalanine to acetophenone in leaves mainly resulted in little occurrence of 1PE in leaves. This study provided an example that differential distribution of some metabolites in plant tissues was not only determined by enzyme(s) in the last step of metabolite formation, but also can be due to substrate availability.

## 1. Introduction

Plants synthesize and emit a large variety of volatile organic compounds, which have important ecological functions such as protective effects against biotic stress and attraction for species-specific pollinators [1,2,3]. In addition, plant volatiles possess potential economic applications including improvement of food storage and flavor, sedation, and improvement of memory [4,5]. Owing to their importance and applications, biosyntheses, spatial and temporal distributions, and regulation mechanisms of formation and emission of plant volatiles have been intensively investigated [6,7]. According to the main metabolic routes, the major classes of plant volatiles can be classified into volatile terpenes, volatile fatty acid derivates, and volatile phenylpropanoids and benzenoids (VPBs) [2]. Many studies have demonstrated that not all plant tissues are equally involved in the production of plant volatiles, and differences within plants are quite common. Plant volatiles from different tissues have different ecological functions. In general, volatiles from vegetative parts, for example leaves, mostly relate to protective effects against biotic stress by deterring herbivores, attracting the enemies of herbivores, and plant-plant or within-plant signaling, whereas volatiles from floral parts are mainly involved in attraction for species-specific pollinators [1,3]. Recently Schiestl analyzed the occurrence, commonness, and evolutionary patterns of the 71 most common ‘floral’ volatile organic compounds in 96 plant families and 87 insect families [8]. It was found that an overlap of 87% in volatiles produced by plants and insects. “Floral” monoterpenes showed strong positive correlation in commonness between plants (both gymnosperms and angiosperms) and herbivores, whereas the commonness of “floral” aromatics (i.e., VPBs) was positively correlated between angiosperms and both pollinators and herbivores. Differential distributions of many “floral” aromatics such as benzylbenzoate, 3,5-dimethoxytoluene, 2-phenylethanol, eugenol, and isoeugenol between leaves and flowers were reported to be due to enzyme(s) or gene(s) in the last step of formation of these volatiles [9,10,11,12].

VPBs containing an aromatic ring are the second most ubiquitous class of plant volatiles, and most VPBs are derived from shikimate via l-phenylalanine (l-Phe) [13]. Isolation and characterization of enzymes and genes involved in the final steps of the biosynthesis of many VPBs (including phenylacetaldehyde, 2-phenylethanol, phenylethylbenzoate, benzaldehyde, benzylalcohol, benzylbenzoate, eugenol, and isoeugenol) have been intensively investigated in plants [14,15]. However, enzyme(s) involved in formation of 1-phenylethanol (1PE) and its spatial distribution remain unknown in plants. In our previous study, we found that 1PE was abundant in flowers of *Camellia sinensis* (tea) but little occurred in tea leaves, which allowed us to use the *C. sinensis* as a model for studying enzymes involved in 1PE formation and its spatial distribution. In addition, we also proposed the pathways of 1PE derived from l-Phe via acetophenone based on the stable isotope labeling technique [16]. In the present study, we attempted to isolate and partially purify enzymes involved in the final step of formation of 1PE from acetophenone. Moreover, we investigated why 1PE differentially accumulated in tea leaf and tea flower.

## 2. Results and Discussion

### 2.1. Tea Flowers Contained Two Types of Enzymes being Responsible for Formations of (R)-1PE and (S)-1PE, Respectively

**Figure 1:**
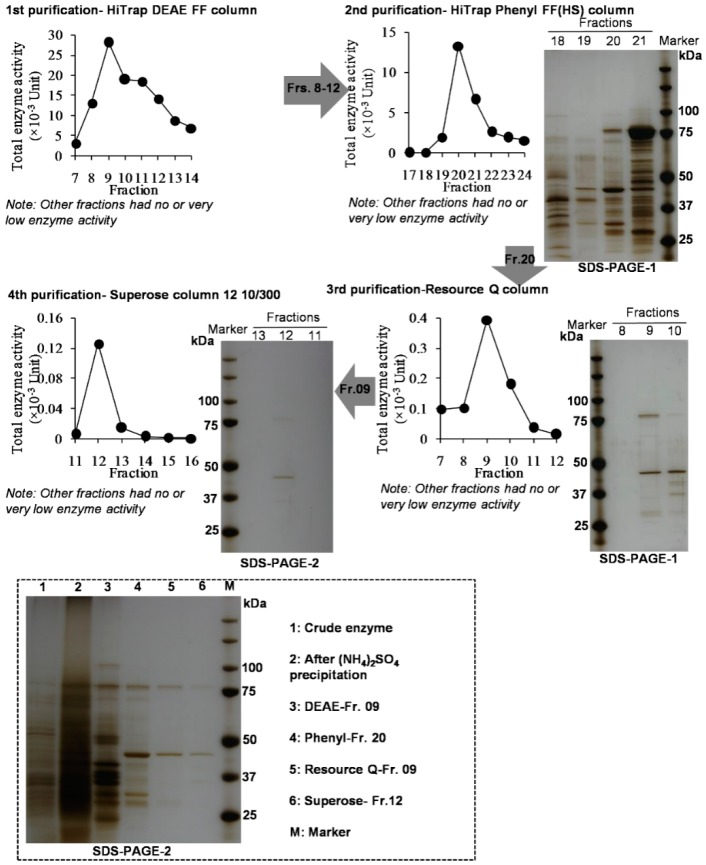
Isolation, partial purification, and SDS-PAGE identification of the enzymes involved in the pathway leading acetophenone to 1PE. Here the unit represented total enzyme activity.

**Figure 2:**
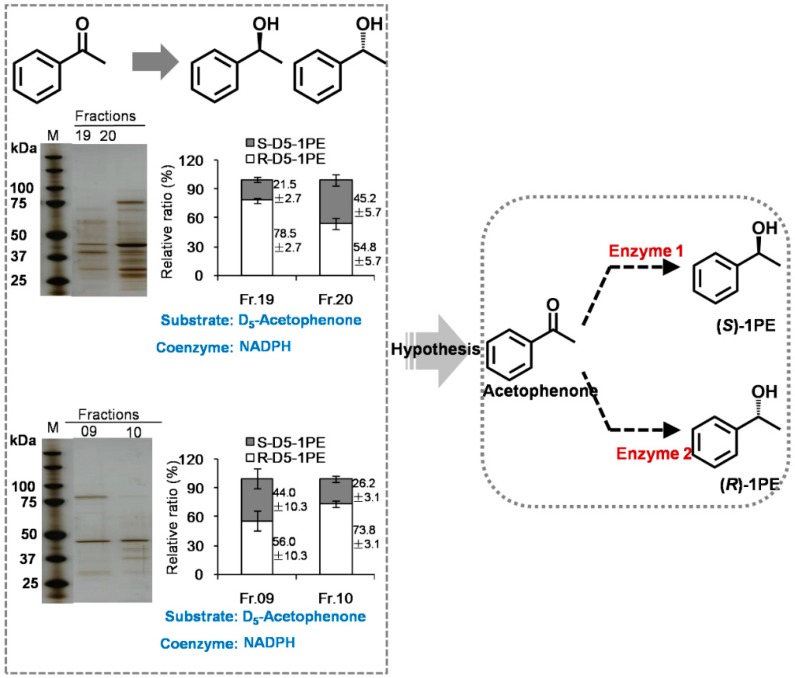
Hypothesis of enzymes involved in the pathways leading from acetophenone to (*R*)-1PE or (*S*)-1PE. The fractions 19 and 20 were from the 2nd purification-HiTrap Phenyl FF(HS) column (See Figure 1). The fractions 09 and 10 were from the 3rd purification-Resource Q column (See Figure 1). Data are expressed as Mean ± S.D. (*n* = 3).

In our previous study, 1PE was identified as a major endogenous volatile compound in tea flowers and mostly occurred in the anthers [16]. (*R*)-1PE was identified as the major isomer of 1PE in tea flowers. In the present study, we investigated the ratio of (*R*)-1PE to (*S*)-1PE in different floral organs. There were significant differences in the ratio of (*R*)-1PE to (*S*)-1PE among floral organs, although (*R*)-1PE was a major 1PE isomer in each floral organ (Table 1). Our previous study had proposed the pathways of 1PE from l-Phe and confirmed that the final step of formation of 1PE was derived from acetophenone [16]. In addition, 1PE could also be transformed to acetophenone, and a short chain dehydrogenase (CsSDR) involved in conversion from (*R*)/(*S*)-1PE to acetophenone has been characterized in tea flowers [17]. However, the enzyme(s) in the pathway leading from acetophenone to (*R*)/(*S*)-1PE is still unknown in plants. Such enzymes have been identified and characterized in some microorganisms such as *Azoarcus sp.* strain EbN1 and *Geotrichum candidum* NBRC 4597 [18,19]. Therefore, initially a homology-based screen approach was employed to search target enzymes in the pathway leading from acetophenone to (*R*)/(*S*)-1PE in tea flowers. By searching in GenBank *C. sinensis* trascriptome database, two genes, whose translated proteins show high similarity to *Azoarcus sp.* strain EbN1 (*S*)-1-PE dehydrogenase [18], were identified. Other two genes, whose translated proteins show high similarity to *Geotrichum candidum* NBRC 4597 acetophenone reductase were found [19]. The four selected genes were subcloned into pET32a expression vector. The recombinant proteins were expressed in *Escherichia coli*. None of these recombinant proteins could transform acetopheneone to (*R*)/(*S*)-1PE. Some of these proteins catalyzed the oxidations of (*R*)/(*S*)-1PE to acetophenone [17] (the cloning data is not shown). Therefore, the desired enzyme(s) were isolated and partially purified from tea flowers by traditional protein chromatography. Using the four different types of chromatography columns, partially purified enzymes were obtained (Figure 1). Furthermore, some isolated active enzymatic fractions transformed [^2^H_5_]acetophenone to more (*R*)-[^2^H_5_]-1PE, whereas some active fractions transformed [^2^H_5_] acetophenone to less (*R*)-[^2^H_5_]-1PE (Figure 2). Here we hypothesize that tea flowers may contain two types of enzymes being responsible for formations of (*R*)-1PE and (*S*)-1PE, respectively.

In general, enzymes within a given class often have highly conserved regions in the protein sequence. Therefore, this method known as homology-based cloning has been widely used to elucidate genes involved in biosynthetic pathways of plant metabolites [20]. However, this method is limited to enzymes with a known conserved sequence. If no consensus sequence for the enzyme has been reported, or the desired enzymes are rare or novel, homology based cloning strategies can not work. In the present study, the *C. sinensis* homologue genes of bacterial acetophenone reductase could not convert acetophenone to (*R*)/(*S*)-1PE. This suggested that the enzymes in the pathway leading from acetophenone to (*R*)/(*S*)-1PE may be a new branch of alcohol dehydrogenase in plants. Although two purified desired enzyme(s) were not successfully obtained by the traditional biochemical approach (Figure 1), two types of enzymes being responsible for formations of (*R*)-1PE and (*S*)-1PE were found to occur in tea flowers (Figure 2). So far, in microorganism, many enzymes were able to conduct the enantioselective reduction of acetophenone. The alcohol dehydrogenases from *Aromatoleum aromaticum* EbN1 and *Geotrichum candidum* NBRC 4597 converted acetophenone to (*S*)-1-PE [19,21]. In *Lactobacillus kefir*, an alcohol dehydrogenase was found to convert acetophenone to (*R*)-1-PE [22]. According to our present study, two enzymes, which were responsible for (*S*)-1-PE and (*R*)-1-PE formation, respectively, may exist in plants.

### 2.2. Differential Distribution of 1PE Between Tea Leaves and Tea Flowers was Due to Availability of Acetophenone

**Figure 3:**
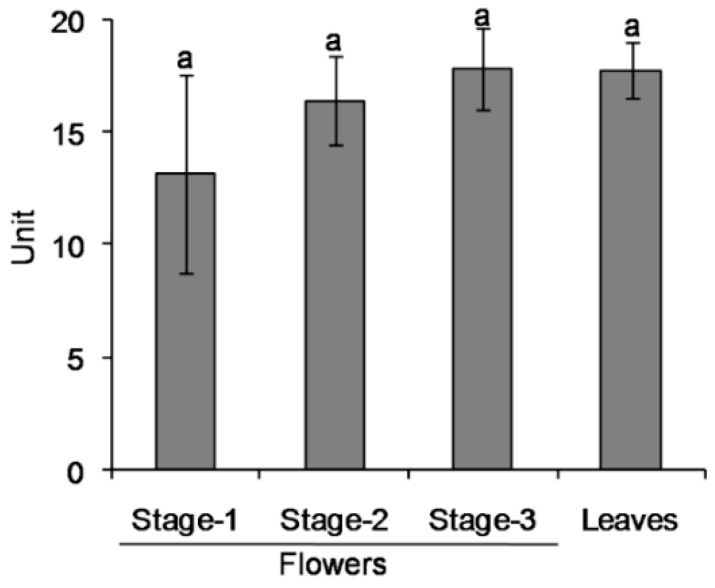
Evaluation of activities of crude enzymes involved in the pathway leading from acetophenone to 1PE from tea flowers and tea leaves. Data are expressed as Mean ± S.D. (*n* = 3). Three groups of tea leaves or tea flowers were used for analysis. Each group contained a mixture of 10 tea flowers or 10 tea leaves (containing one bud and three leaves). Means with same letters are no significant differences (*p* > 0.05). Here the unit represented enzyme specific activity.

**Figure 4:**
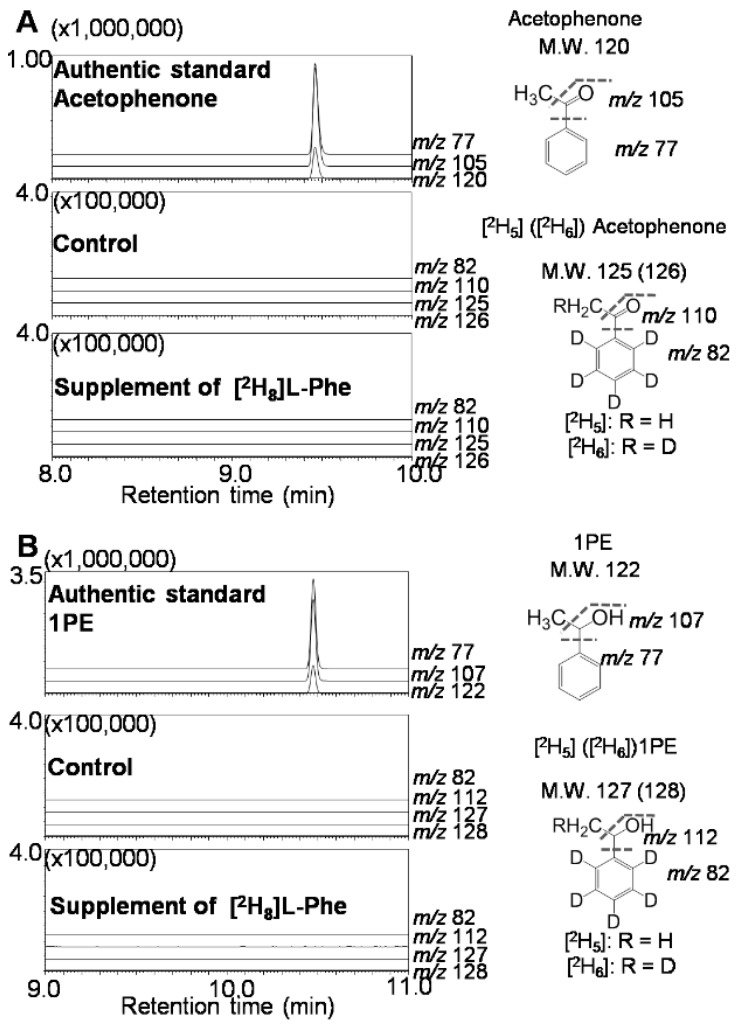
Mass chromatograms of endogenous labeled acetophenone (**A**) and 1PE (**B**) in tea leaves supplied with l-[^2^H_8_]Phe. [M], [M − CH_3_], and [M − OCCH_3_] are characteristic ions of nonlabeled acetophenone, and [M], [M − CH_3_], and [M − OHCHCH_3_] are characteristic ions of nonlabeled 1PE in GC-MS analysis. The fragments of labeled acetophenone and 1PE were assigned based on the pattern of nonlabeled acetophenone and 1PE.

1PE was abundant in tea flowers, but not in tea leaves [16]. To probe into the reason why 1PE differentially accumulated in tea leaves and tea flowers, we investigated whether it was due to the different distribution of enzymes involved in the pathway leading from acetophenone to 1PE in leaves and flowers. Surprisingly, tea leaves had equivalent activity of the crude enzyme with tea flowers at each stage (Figure 3), suggesting that the enzyme(s) in the pathway leading from acetophenone to 1PE were present to a similar degree in tea leaves. To further investigate whether the pathway upstream of 1PE affects the differential distribution of 1PE, labeled l-Phe was supplied to tea leaves. The labeled acetophenone and 1PE was not detected in tea leaves (Figure 4), while with supplement of labeled l-Phe to tea flowers, labeled acetophenone and 1PE could be clearly detected [16]. To exclude possible effects of differential absorptions of labeled l-Phe into tea flowers and tea leaves, we investigated occurrences of labeled l-Phe in the two tissues. In both tea leaves and tea flowers, high concentrations of l-[^2^H_8_]Phe could be detected (data not shown), suggesting that the two tissues could absorb l-[^2^H_8_]Phe during the feeding process. In addition, in tea leaves supplied with l-[^2^H_8_]Phe, [^2^H_8_]-2-phenylethanol, which is an aroma compound derived from l-Phe, was detected (data not shown). These observations indicated that l-[^2^H_8_]Phe was able to be absorbed into tea leaves and metabolized to other compounds. However, weak transformation from l-Phe to acetophenone in leaves mainly resulted in trace occurrence of 1PE in tea leaves.

**Figure 5:**
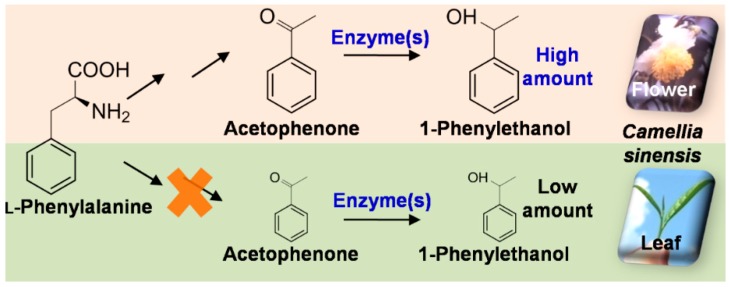
Proposed schematic model of differential accumulation of 1PE in flowers and leaves of tea (*Camellia sinensis*) plants.

The present study confirmed differential distribution of some volatile compound such as 1PE between leaves and flowers could also be determined by substrate availability (Figure 5). Many VPBs such as 2-phenylethanol, benzyl alcohol, benzaldehyde, and methyl salicylate, which are derived from l-Phe, occur in tea leaves in certain amounts [23,24,25]. Tea flowers also had these VPBs with equivalent levels of tea leaves [17]. In contrast, acetophenone and 1PE, which are also derived from l-Phe, accumulated differentially in leaf and flower. Our previous study proposed a pathway leading from l-Phe to 1PE via *trans*-cinnamic acid, 3-hydroxy-3-phenylpropionic acid, and acetophenone [16]. Further studies on enzymes and genes involved in the pathway leading from *trans*-cinnamic acid to acetophenone may help us to find out the key enzyme(s), which result in occurrence of 1PE in tea leaves with little amount.

## 3. Materials and Methods

### 3.1. Plant Materials

The flowers and leaves of *C. sinensis* var. Jinxuan were collected from tea fields at the South China Agricultural University (Guangzhou, China) between October and November. As described in our previous studies [16,17,26,27], floral development was divided into 3 stages: at stage 1 the flower buds are closed, at stage 2 the flower is half open, and at stage 3 the flower is fully open. In addition, the flowers at stage 3 were divided into four parts including petals, filaments, anthers, and receptacles. All tissues were frozen in liquid nitrogen and stored at −80 °C.

### 3.2. Determination of Stereochemistry of 1PE from Different Tissues of Tea Flowers

To analyze the ratio of (*R*)-1PE to (*S*)-1PE in each floral organ, finely powdered floral tissues (500 mg fresh weight) were extracted overnight in the dark with 1 mL of hexane:ethyl acetate (1:1). The extract was then dried over anhydrous sodium sulfate and 1 μL of the filtrate was subjected to GC-MS QP5000 (Shimadzu, Tokyo, Japan) equipped with an InertCap CHIRAMIX column (30 m × 0.25 mm × 0.25 μm, GL Sciences, Inc., Torrence, CA, USA). Helium was used as the carrier gas at a flow rate of 1.2 mL/min. The temperatures were 40 °C as an initial temperature, a ramp of 4 °C/min to 180 °C, then 180 °C for 40 min. MS was performed in full scan mode (mass range *m*/*z* 70–200) and SIM mode.

### 3.3. Extraction of Crude Enzymes Involved in the Pathway Leading from Acetophenone to 1PE from Tea Flowers and Tea Leaves

Finely powdered plant tissues (350 mg) were added with 0.1 g of polyvinylpolypyrrolidone (PVPP), homogenized in 3.5 mL of cold buffer A (100 mM pH 7.0 potassium phosphate buffer containing 1% glycerol, 1 mM ethylenediamine tetraacetic acid (EDTA), and 0.2% 3-[(3-cholamidopropyl)-dimethylamino]-1-propanesulfonate (CHAPS) under ice, and centrifugated (26,740 *g*, 4 °C, 20 min). The supernatant was centrifugated again (26,740 *g*, 4 °C, 20 min) to remove suspended substances, then loaded on a PD-10 desalting column (GE Healthcare Bio-Sciences, Piscataway, NJ, USA), and eluted using 10 mM potassium phosphate buffer (pH 7.0) containing 0.1% glycerol and 0.1 mM EDTA. The eluate was used as a crude enzyme solution.

### 3.4. Assay of the Crude Enzymes from Tea Flowers and Tea Leaves

The reaction mixture contained 200 μL enzyme solution, 50 μL substrate (0.4 μmol labeled [^2^H_5_]ring-acetophenone (98%, Cambridge Isotope Laboratories, Inc., Andover, MA, USA), 50 μL coenzyme (1.2 μmol NADPH), and 100 μL of 100 mM potassium phosphate buffer (pH 5.3). The reaction was incubated at 40 °C for 60 min. Afterwards, 5 nmol of ethyl *n*-decanoate as an internal standard was added. The reaction products were extracted with 0.4 mL of hexane:ethyl acetate (1:1), and centrifugated (10,000 *g*, 4 °C, 3 min). The supernatant was dried over anhydrous sodium sulfate. Samples were then analyzed by GC-MS. The temperature of the injector was 230 °C. The GC was equipped with a capillary SUPELCOWAX^TM^ 10 column (Supelco Inc., Bellefonte, PA, USA, 30 m × 0.25 mm I.D., 0.25 μm film thickness). Helium was used as a carrier gas at a flow rate of 1.6 mL/min. The GC oven was maintained at 60 °C for 3 min. The temperature of the oven was programmed at 40 °C/min to 180 °C and then at 10 °C/min to 240 °C, and kept at this temperature for 3 min. The mass spectrometry was operated by the full scan mode (mass range *m*/*z* 70–200).

Enzyme activity was defined as the formed [^2^H_5_]ring-1PE amount in the standard enzyme reaction system. The formed [^2^H_5_]ring-1PE amount was evaluated by peak area ratio of analyte to internal standard (ethyl *n*-decanoate). Peak areas of the internal standard were calculated as summation of *m*/*z* 88 and *m*/*z* 101. [^2^H_5_]ring-1PE peak areas were calculated as summation of *m*/*z* 84, *m*/*z* 112, and *m*/*z* 127. Enzyme specific activity was defined as enzyme activity/mg protein in crude enzyme solutions.

### 3.5. Isolation and Partial Purification of the Enzymes Involved in the Pathway Leading from Acetophenone to 1PE from Tea Flowers

Finely powdered plant tissues (20 g) were homogenized on ice with 6 g PVPP, 0.4 g CHAPS, and 200 mL cold 100 mM potassium phosphate buffer (pH 7.0) containing 1% glycerol, 1 mM EDTA, 2.5 mM dl-dithiothreitol (DTT), and 0.25 mM 4-(2-aminoethyl)-benzenesulfonyl fluoride. The homogenized solution was centrifugated (26,740 *g*, 4 °C, 20 min). Six g PVPP was added to the supernatant and then centrifugated (26,740 *g*, 4 °C, 20 min) again. The resultant supernatant was precipitated by 50% ammonium sulfate, and kept for 2 h at 4 °C. Afterwards the supernatant was precipitated by 60% ammonium sulfate, kept for 2 h at 4 °C, and then centrifuged (26,740 *g*, 4 °C, 20 min). The precipitate was dissolved in cold 10 mM pH 8.0 potassium phosphate buffer containing 1% glycerol, 0.1 mM EDTA , 0.25 mM DTT , and 0.02% CHAPS (buffer A). The enzyme solution was filtered through a nylon filter to remove the suspended substances, desalted by dialysis membrane (10 h, 4 °C), and then sequentially subjected to HPLC by HiTrap DEAE FF column, HiTrap Phenyl FF (HS) column, Resource-Q column, and Superose column, successively. The HPLC conditions are shown below. (1) The 1st purification: column: HiTrap DEAE FF column (5 mL× 2); flow rate: 1 mL/min; column temperature: 20 °C; mobile phase A: buffer A (pH 8.0, as shown above); mobile phase B: 1 M NaCl in mobile phase A; gradient: from 0 min to 20 min, mobile phase B maintained at 0%; from 20 min to 100 min, mobile phase B increased from 0% to 25%; from 100 min to 120 min, mobile phase B increased from 25% to 100%; collection: 5 mL/fraction; (2) The 2nd purification: column: HiTrap Phenyl FF (HS) column (5 mL× 2); flow rate: 1 mL/min; column temperature: 20 °C; mobile phase A: buffer A (pH 8.0, as shown above); mobile phase B: 1.5 M (NH_4_)_2_SO_4_ in mobile phase A; gradient: from 0 min to 20 min, mobile phase B maintained at 100%; from 20 min to 120 min, mobile phase B decreased from 100% to 0%; from 120 min to 140 min, mobile phase B maintained at 0%; collection: 5 mL/fraction; (3) The 3rd purification: column: Resource Q column (1 mL); flow rate: 0.5 mL/min; column temperature: 20 °C; mobile phase A: buffer A (pH 8.0, as shown above); mobile phase B: 1 M NaCl in mobile phase A; gradient: from 0 min to 10 min, mobile phase B maintained at 0%; from 10 min to 30 min, mobile phase B increased from 0% to 25%; from 30 min to 40 min, mobile phase B increased from 25% to 100%; collection: 1 mL/fraction; (4) The 4th purification: column: Superose column 12 10/300 (24 mL); flow rate: 0.5 mL/min; column temperature: room temperature; mobile phase: 20 mM pH 7.0 potassium phosphate buffer containing 0.1 M NaCl, 0.1 mM EDTA, 0.25 mM DTT, and 0.02% CHAPS; 0–60 min elution; collection: 1 mL/fraction. All the obtained fractions were subjected to the enzyme assay and the enzymatic product 1PE was analyzed by GC-MS (Section 3.4). In addition, the stereochemistry of 1PE was determined as described in Section 3.2.

### 3.6. Supplement of Stable Isotope-Labeled Compound to Tea Leaves and Identification of Products

Tea leaves (containing one bud and three leaves) were placed in one of the following solutions: (1) water (as control); (2) 12 mM l a,b,b,2,3,4,5,6-^2^H_8_]Phe (l-[^2^H_8_]Phe, deuterium atom ≥ 98%, Cambridge Isotope Laboratories, Inc.). After 18 h treatment, to identify endogenous volatile products, finely powdered floral tissues (500 mg fresh weight) were extracted with 2 mL of hexane:ethyl acetate (1:1) for 7 h in the dark. The extract was then filtered through a short plug of anhydrous sodium sulfate and 1 μL of the filtrate was subjected to GC-MS analysis. The injector temperature was 230 °C, splitless mode was used with a splitless time of 1 min, and helium was the carrier gas with a velocity 1.6 mL/min. The SUPELCOWAX^TM^ 10 column (Supelco Inc., 30 m × 0.25 mm × 0.25 μm) was used with an initial temperature of 60 °C, a ramp of 20 °C/min to 180 °C, and then 10 °C/min to 240 °C, and hold at 240 °C for 20 min. The MS analyses were carried out in full scan mode (mass range *m*/*z* 44–200).

### 3.7. Statistical Analysis

One-way ANOVA was used to determine the differences. A probability level of 5% (*p* ≤ 0.05) was considered as significant level. One-way ANOVA was processed by using the SPSS 11.5 program (SPSS Inc., Chicago, IL, USA).

## 4. Conclusions

Our present study proposed that two types of enzymes were responsible for the formation of (*R*)-1PE and (*S*)-1PE from acetophenone, respectively, which could be confirmed in tea flowers by direct evidences. Furthermore, this study provided an example that differential distribution of some VPB between leaves and flowers could also be determined by substrate availability and not due to the enzyme(s) in the final step of formation of the VPB (Figure 5). The information contributes to our understanding of biosynthesis of VPBs in plants and their distributions within plants. In addition, there are abundant and non-utilized resource of tea flowers, for example over 1.8 billion kilograms of tea flowers are available annually in China [26]. 1PE is used as fragrance in the food flavor and cosmetic industries because of its mild floral odour and as an intermediate in pharmaceutical industry. This study could also provide essential information for future application of tea flowers in the food flavor and cosmetic industries.

## Figures and Tables

**Table 1:** Ratios of (*R*)- to (*S*)-1PE in the endogenous 1PE in different floral organs.

Floral Organs	(*R*)-1PE	(*S*)-1PE
Filaments	97.2 ± 0.3 ^a^	2.8 ± 0.3 ^a^
Anthers	92.6 ± 0.1 ^bc^	7.4 ± 0.1 ^bc^
Petals	90.1 ± 1.8 ^c^	9.9 ± 1.8 ^c^
Receptacles	86.0 ± 1.9 ^d^	14.0 ± 1.9 ^d^

The data are expressed as mean ± S.D. (*n* = 3). Three groups of floral organs were used for analysis. Each group contained floral organs from a mixture of 15 tea flowers. Different mean ± S.D. with different letters in the same column are significantly different from each other (*p* ≤0.05).

## Abbreviations

CHAPS	3-[(3-Cholamidopropyl)-dimethylamino]-1-propanesulfonate
DTT	dl-Dithiothreitol
EDTA	Ethylenediamine tetraacetic acid
1PE	1-Phenylethanol
l-Phe	l-Phenylalanine
PVPP	Polyvinylpolypyrrolidone
VPBs	Volatile phenylpropanoids and benzenoids
